# The effects of meldonium on the acute ischemia/reperfusion liver injury in rats

**DOI:** 10.1038/s41598-020-80011-y

**Published:** 2021-01-14

**Authors:** Siniša Đurašević, Maja Stojković, Jelena Sopta, Slađan Pavlović, Slavica Borković-Mitić, Anđelija Ivanović, Nebojša Jasnić, Tomislav Tosti, Saša Đurović, Jelena Đorđević, Zoran Todorović

**Affiliations:** 1 Faculty of Biology, University of Belgrade, 16 Studentski Trg, 11000 Belgrade, Republic of Serbia; 2 Faculty of Medicine, University of Belgrade, Belgrade, Republic of Serbia; 3 Institute for Biological Research “Siniša Stanković” – National Institute of Republic of Serbia, University of Belgrade, Belgrade, Republic of Serbia; 4 Faculty of Chemistry, University of Belgrade, Belgrade, Republic of Serbia; 5 Institute of General and Physical Chemistry, University of Belgrade, Belgrade, Republic of Serbia; 6 University Medical Centre “Bežanijska Kosa”, University of Belgrade, Belgrade, Republic of Serbia

**Keywords:** Liver, Drug discovery, Physiology

## Abstract

Acute ischemia/reperfusion (I/R) liver injury is a clinical condition challenging to treat. Meldonium is an anti-ischemic agent that shifts energy production from fatty acid oxidation to less oxygen-consuming glycolysis. Thus, we investigated the effects of a 4-week meldonium pre-treatment (300 mg/kg b.m./day) on the acute I/R liver injury in Wistar strain male rats. Our results showed that meldonium ameliorates I/R-induced liver inflammation and injury, as confirmed by liver histology, and by attenuation of serum alanine- and aspartate aminotransferase activity, serum and liver high mobility group box 1 protein expression, and liver expression of Bax/Bcl2, haptoglobin, and the phosphorylated nuclear factor kappa-light-chain-enhancer of activated B cells. Through the increased hepatic activation of the nuclear factor erythroid 2-related factor 2, meldonium improves the antioxidative defence in the liver of animals subjected to I/R, as proved by an increase in serum and liver ascorbic/dehydroascorbic acid ratio, hepatic haem oxygenase 1 expression, glutathione and free thiol groups content, and hepatic copper-zinc superoxide dismutase, manganese superoxide dismutase, catalase, glutathione peroxidase, and glutathione reductase activity. Based on our results, it can be concluded that meldonium represent a protective agent against I/R-induced liver injury, with a clinical significance in surgical procedures.

## Introduction

Acute ischemia/reperfusion (I/R) refers to a temporary abolishment of blood flow during surgical interventions, followed by its re-establishment once an intervention is finished^1^. I/R damages affected tissue through numerous pathological processes, such as inflammation, oxidative stress, disturbance of cellular signalling pathways, and cell apoptosis and necrosis^2^. The hepatic I/R molecular pathways begin with increased radical oxygen species (ROS) formation during the ischemic phase. Cellular damage induced by ROS causes a release of Damage-Associated Molecular Pattern Molecules (DAMPs), such as high mobility group Box 1 protein (HMGB1), which is known to induce inflammation by activating the nuclear factor kappa-light-chain-enhancer of activated B cells (NF-κB p65) pathway^3^. The resulting release of inflammatory cytokines and chemokines leads to the consequent activation of pro-inflammatory neutrophils, which further amplify the tissue destruction^4^.

There are several possible therapeutic options for I/R liver injury, such as organ preservation, inhibition of ROS formation, or the modulation of immune system activation^5^. While the organ preservation strategy showed the most promising results^6^, the use of TNF inhibitors^7^, corticosteroids^8^, or free-radical scavengers such as *N*-acetyl cysteine^9^, has been mostly unsuccessful, or with limited success. In our previous work in the model of renal I/R, we showed that meldonium, a clinical drug used to treat myocardial and cerebral ischemic conditions^10^, exhibits anti-inflammatory properties^11^. It inhibits synthesis and transport of carnitine, disturbing transport of cytosolic long-chain fatty acids (FAs) into mitochondria, and redirecting it to peroxisomes^12^. In this way, meldonium protects mitochondria against the accumulation of toxic long-chain FAs intermediates^13^, and shifts energy production to glycolysis, which is less oxygen-demanding in comparison with the FAs oxidation, and thus more favourable under ischemic conditions.

For these reasons, the protective effect of a four-week meldonium pre-treatment in a dose of 300 mg/kg b.m./day was investigated in a model of rat hepatic I/R. This dose was chosen because it is similar to the clinical human use^14,15^, and within the range of previously studied animal doses of meldonium^16^, where it proved to be both highly effective and safe. As a general marker of meldonium metabolic action, liver concentration of glucose, lactic acid, carnitine, FA, and glycerol were measured. The extent of liver injury was assessed by a tissue histology, serum alanine aminotransferase (ALT), aspartate aminotransferase (AST), and alkaline phosphatase (ALP) activity, NF-κB p65 activation by phosphorylation, and expression of NF-kB p65-regulated acute-phase protein haptoglobin (Hp). The ratio of Bax and Bcl-2 in the liver and the serum and liver HMGB1 levels were measured as the markers of apoptotic and necrotic cell death. The liver antioxidant defence was investigated by monitoring the activation of nuclear factor erythroid 2-related factor 2 (Nrf2) by phosphorylation, and expression of Nrf2-regulated antioxidative enzyme haem oxygenase 1 (HO-1), and by measuring the activity of the copper-zinc superoxide dismutase (CuZnSOD), manganese superoxide dismutase (MnSOD), catalase (CAT), glutathione peroxidase (GSH-Px), glutathione reductase (GR), and glutathione S-transferase (GST), free sulfhydryl groups (SH) and glutathione (GSH) concentration, and the level of lipid peroxidation (thiobarbituric acid reactive substances, TBARS). Ascorbic acid is a well-known effector of the antioxidative system, so our experiment also included the determination of serum, adrenal gland, and liver ascorbic acid (AA) and dehydroascorbic acid (DHA) concentration. Since the link between ascorbic acid and catecholamine production in the adrenal glands is well known^17,18^, we also determined the noradrenaline (NA) and adrenaline (AD) concentrations in the adrenal glands.

## Results and discussion

Our finding that I/R increases hepatic lactic acid concentration for 50% (Table 1) suggests that, rather than oxidative phosphorylation meeting the ATP demand, glycolysis hastened ATP production at the substrate-level during I/R. In aerobic conditions, pyruvate, as the end product of glycolysis, enters the Krebs cycle avoiding the lactate production^19^. However, under anaerobic conditions, as is the case with I/R, it is converted by lactate dehydrogenase to lactic acid^20^. The fact that hepatic glucose content was unaffected by I/R (Table 1) is probably the result of the short I/R duration, and otherwise high liver glucose content.

**Table 1:** Liver carnitine (μg/g wet tissue mass), and glucose, lactic acid, triglycerides, and glycerol concentration (μg/mg wet tissue mass).

	S	S + M	I/R	I/R + M
Glucose	2.18 ± 0.11	1.19 ± 0.13^a^	2.23 ± 0.15	1.51 ± 0.18^ab^
Lactic acid	25.39 ± 0.83	14.46 ± 0.84^a^	36.97 ± 0.93^a^	25.71 ± 1.00^b^
Carnitine	149.20 ± 3.95	91.58 ± 4.55^a^	498.69 ± 11.96^a^	338.12 ± 16.72^ab^
Triglycerides	6.44 ± 0.09	6.04 ± 0.04	3.25 ± 0.10^a^	5.86 ± 0.03^ab^
Glycerol	0.049 ± 0.001	0.036 ± 0.001^a^	0.152 ± 0.002^a^	0.052 ± 0.001^b^

Experimental group abbreviations: *S* sham-operated rat group, *S + M* sham-operated + meldonium rat group, *I/R* ischemia/reperfusion rat group, *I/R + M* ischemia/reperfusion + meldonium rat group. The number of animals per experimental group: n = 8. The data are given as mean ± standard error of the mean. Minimal significant level: p < 0.05. Significantly different: ^a^in respect to S group; ^b^in respect to I/R group.

Carnitine is essential for mitochondrial β-oxidation, since long-chain fatty acyl-CoA needs to be converted to fatty acyl-carnitine by carnitine palmitoyltransferase I for transport over the outer mitochondrial membrane, and further transport by the carnitine-acylcarnitine translocase across the inner membrane. Once inside the mitochondrial matrix, fatty acyl-carnitine is re-converted to fatty acyl-CoA by carnitine palmitoyltransferase II, becoming ready for the ATP production through β-oxidation. Thus, the I/R-induced decrease in liver triglycerides content for 50%, along with the increase of hepatic glycerol, FAs, and carnitine concentration (Tables 1 and 2) implicate an increase in liver lipolysis and FAs β oxidation. This is probably a compensatory reaction against impaired ATP production due to the substrate-level glycolysis, and the same effect has been described in the ischemic/reperfused heart^21,22^.

**Table 2:** Hepatic fatty acids concentration (µg/mg tissue).

	S	S + M	I/R	I/R + M
C14:0	0.086 ± 0.007	0.030 ± 0.003^a^	0.125 ± 0.005^a^	0.067 ± 0.009^b^
C15:0	0.064 ± 0.003	0.037 ± 0.002^a^	0.152 ± 0.015^a^	0.072 ± 0.008^b^
C16:0	5.221 ± 0.155	2.114 ± 0.150^a^	10.585 ± 0.445^a^	4.766 ± 0.311^b^
C16:1	0.444 ± 0.021	0.149 ± 0.010^a^	0.631 ± 0.033^a^	0.262 ± 0.012^ab^
C17:0	0.312 ± 0.016	0.139 ± 0.007^a^	0.708 ± 0.034^a^	0.304 ± 0.020^b^
C17:1	0.039 ± 0.003	0.020 ± 0.001^a^	0.076 ± 0.006^a^	0.035 ± 0.003^b^
C18:0	5.160 ± 0.136	2.109 ± 0.102^a^	11.730 ± 0.477^a^	4.935 ± 0.239^b^
C18:1 *cis*	0.828 ± 0.041	0.364 ± 0.025^a^	1.717 ± 0.128^a^	0.899 ± 0.049^b^
C18:1 *trans*	2.121 ± 0.208	0.667 ± 0.038^a^	2.933 ± 0.114^a^	1.484 ± 0.149^ab^
C18:2 c + t	4.900 ± 0.217	1.895 ± 0.193^a^	9.482 ± 0.386^a^	4.301 ± 0.358^b^
C18:3 n3	0.054 ± 0.004	0.018 ± 0.001^a^	0.078 ± 0.005^a^	0.047 ± 0.002^b^
C20:1	0.019 ± 0.001	0.012 ± 0.001^a^	0.037 ± 0.005^a^	0.021 ± 0.001^b^
C20:2	0.117 ± 0.005	0.045 ± 0.003^a^	0.263 ± 0.015^a^	0.128 ± 0.006^b^
C20:3 n6	0.072 ± 0.003	0.029 ± 0.003^a^	0.156 ± 0.007^a^	0.050 ± 0.004^ab^
C20:3 n3	5.125 ± 0.083	2.406 ± 0.142^a^	12.578 ± 0.547^a^	5.455 ± 0.249^b^
C22:6 + C22:1	0.819 ± 0.029	0.393 ± 0.020^a^	1.766 ± 0.066^a^	0.827 ± 0.035^b^

Experimental group abbreviations: *S* sham-operated rat group, *S + M* sham-operated + meldonium rat group, *I/R* ischemia/reperfusion rat group, *I/R + M* ischemia/reperfusion + meldonium rat group. The number of animals per experimental group: n = 8. The data are given as mean ± standard error of the mean. Minimal significant level: p < 0.05. Significantly different: ^a^in respect to S group; ^b^in respect to I/R group.

Being a γ-butyrobetaine analogue, meldonium inhibits the activity of γ-butyrobetaine hydroxylase, the enzyme that catalyses the formation of l-carnitine^23^, and the l-carnitine renal re-absorption^24^. Our results show that meldonium decreases liver carnitine, glycerol, and FAs concentration in both sham and I/R groups of rats, and increases triglycerides content in I/R operated animals (Tables 1 and 2). In addition, the I/R-induced liver lactate concentration increase was reduced by meldonium for 30%, and that the same effect was observed in sham-operated animals (50% decrease in comparison to the S group). It should be noted that meldonium also decreases glucose content in sham and I/R operated animals (Table 1), which is consistent with the literature data showing an increase in glucose uptake and a decrease in the lactate concentration in the mouse heart treated with meldonium^25^. In that sense, our results implicate that meldonium decreases liver lipolysis and FAs β oxidation, and stimulates aerobic oxidation of glucose as an oxygen-sparing mechanism for ATP production under ischemic conditions.

**Figure 1:**
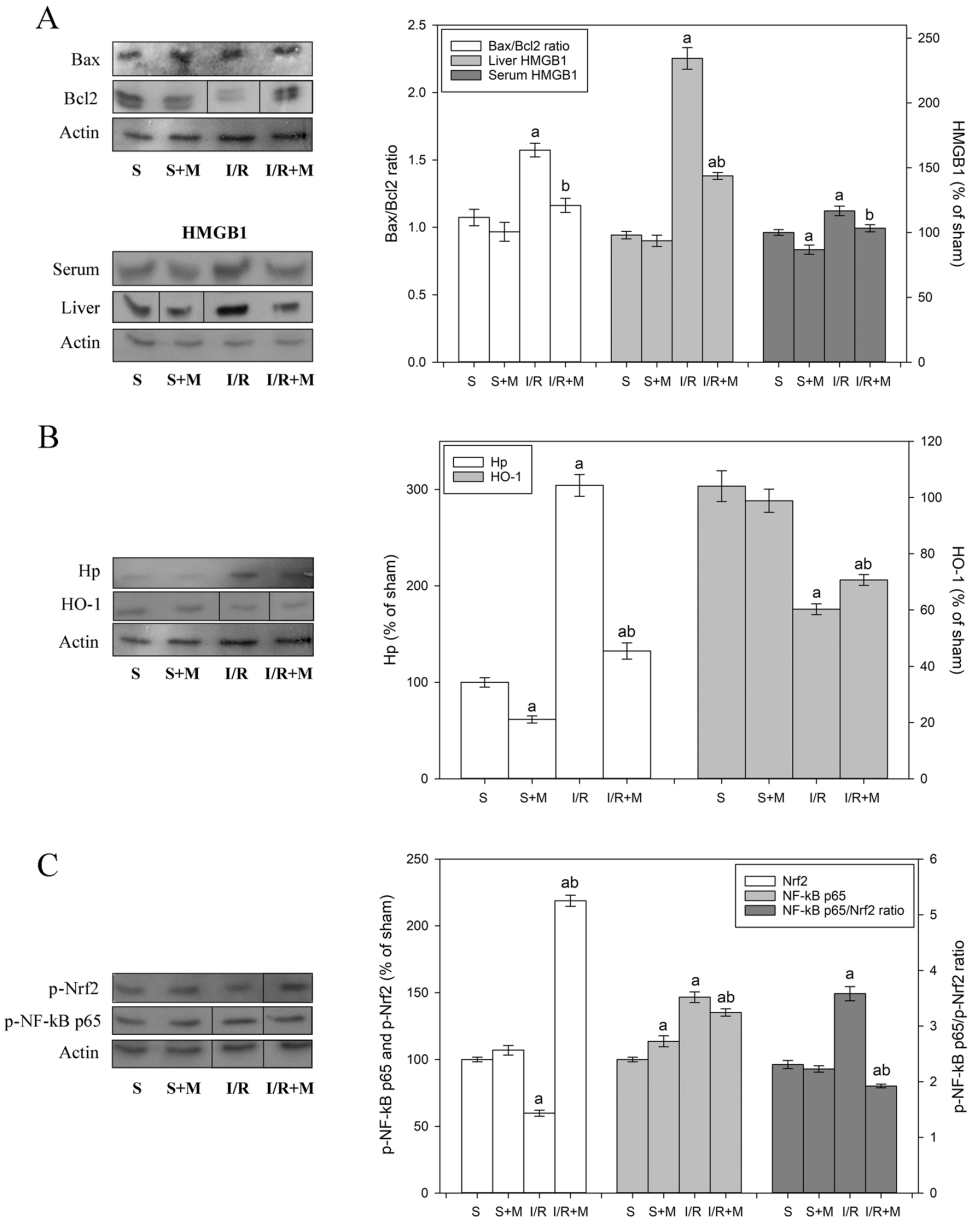
Representative immunoblots of protein expression levels: (**A**) liver Bax/Bcl-2 ratio, and liver and serum HMGB1 expression; (**B**) liver Hp and HO-1 expression; and (**C**) liver p-Nrf2 and p-NF-κB p65 expression, and p-NF-κB p65/p-Nrf2 ratio. β actin was used as a loading control. The blots cropped from different gels are delineated with the black line (see Supplementary Information). Experimental group abbreviations: *S* sham-operated rat group, *S + M* sham-operated + meldonium rat group, *I/R* ischemia/reperfusion rat group, *I/R + M* ischemia/reperfusion + meldonium rat group. The number of animals per experimental group: n = 8. Data are given as mean ± standard error of the mean. Minimal significant level: p < 0.05. Significantly different: ^a^in respect to S group; ^b^in respect to I/R group.

The restoration of normal oxygen level during reperfusion causes a release of cytokines and chemokines from activated tissue-resident macrophages and infiltration of pro-inflammatory neutrophils^26^. By releasing oxidants and proteases, the recruited neutrophils damage hepatocytes and lead to the massive apoptotic and necrotic cell death response^27^. It has been suggested that a delicate interplay between anti- and pro-apoptotic members of the Bcl2 family of proteins is necessary for the repair of damaged cells after an ischemic insult^28^, and that overexpression of anti‐apoptotic Bcl2 can block both apoptosis and necrosis^29^, and protect ischemic tissue against I/R-induced oxidative stress^30^. Our finding that I/R increases the liver Bax/Bcl2 ratio for almost 50% and that meldonium reduces this increase by returning the value to the control level (Fig. 1A), points to the acceleration of pro-apoptotic events during I/R, and its effective reduction by meldonium.

Apoptosis is an energy-requiring process, so the impaired ATP production in I/R may cause failure in the apoptosis induction, and the development of necrosis followed by the uncontrolled release of DAMPs into the extracellular space^31^. One of these molecules, the HMGB1, diffuses out of the stressed, damaged, or dying cells, which is why it serves as a necrotic marker^32^. Our results show that I/R and meldonium change HMGB1 expression in a manner similar to Bax/Bcl2: while I/R increases the liver and serum levels of HMGB1 (twofold and 20%, respectively), concurrent meldonium pre-treatment reduces it for 30% and 20%, respectively (Fig. 1A). The fact that I/R-induced HMGB1 release was more pronounced in the liver than in serum speaks of the tissue localisation of this process. This is important since HMGB1 further propagates inflammation upon release in serum by activating the NF-κB pathway through binding to TLR2, TLR4, TLR9, and the advanced glycation end products (RAGE) receptors of leukocytes or endothelial cells^33^. It can be said that these results prove that meldonium protects not only against the I/R-induced necrosis but also inflammation^34^.

Haptoglobin is the primary haemoglobin (Hb) binding plasma protein which attenuates the adverse effects of extracellular Hb. The cellular receptor of the Hb-Hp complex is the monocyte/macrophage CD163 scavenger receptor. After the binding to CD163, cellular internalisation of the complex leads to the effective Hb neutralisation^35^. Based on our results, I/R causes a threefold increase in the liver Hp level, which concurrent meldonium treatment reduced back to the control level (Fig. 1B). Since the same effect was also present in sham-operated animals (40% decrease in comparison to control group, Fig. 1B), it can be concluded that meldonium increases liver-adaptive responses to tissue injury through enhanced Hb sequestration and clearance.

Another factor that protects against tissue injury is HO-1, an enzyme that plays a vital role in the catabolism of haem^36^. It has been shown that HO-1^−/−^ knockdown mice develop the progressive inflammatory disease followed by a robust increase in pro-inflammatory cytokines^37^, and that overexpression of HO-1 attenuates cellular damage and reduces apoptosis in the liver^38^. The HO-1 promoter contains binding sites for various transcription factors, including phosphorylated Nrf2^39^. Our finding that I/R decreases hepatic levels of p-Nrf2 and HO-1 by about 40%, which concurrent meldonium pre-treatment increases for a fivefold and 20%, respectively (Fig. 1B,C), points to a new role of the Nrf2/HO-1 signalling pathway in meldonium protection against I/R-induced liver injury.

Initially identified as a DNA binding protein in activated B cells^40^, the NF-κB modulates the gene expression in diverse cellular processes, such as stress responses to a variety of noxious stimuli, or cell proliferation and apoptosis^41^. Accordingly, inflammatory signals activate NF-kB p65 by phosphorylation^42^, causing its nuclear translocation and transactivation of target genes, including the gene for Hp^43^. Our results show that I/R increases the hepatic expression of phosphorylated NF-κB p65 (Fig. 1C), which is in line with the literature data showing time-dependent activation of NF-κB p65 after the initiation of reperfusion^44^. While in S + M group of animals meldonium does not change hepatic Nrf2 phosphorylation, in I/R + M group, there is a 2.5-fold increase (Fig. 1C). Thus, as a result of simultaneous NF-κB p65 and Nrf2 activation changes, the p-NF-κB p65/p-Nrf2 ratio remained unchanged in the S + M group, increased by 70% in the I/R group, and strongly decreased in the I/R + M group. These results point to a new hepatoprotective role of meldonium mediated through the p-NF-κB p65/p-Nrf2 pathways interplay, in accordance with the literature data showing beneficial effects of NF-κB expression decrease in ischemic liver^45,46^.

It is known that Nrf2 increases the expression of many antioxidative enzymes by binding to antioxidant response elements^47^, such as CuZnSOD, MnSOD, GSH-Px, GST, and CAT^48^, Nrf2 also controls the expression of the catalytic and the modifier subunits of the glutamate-cysteine ligase complex that catalyses the reaction of glutamate with cysteine, the rate-limiting step in the synthesis of GSH^49^. Our results show that the p-Nrf2 expression decrease in I/R group (Fig. 1C) was followed by a 30%–50% decrease in hepatic CuZnSOD, MnSOD, and GSH-Px activity, and a 2.5-fold decrease in the liver GSH content (Table 3). Unexpectedly, despite the I/R-induced p-Nrf2 expression decrease, the CAT activity remained unchanged in comparison with the sham-operated controls (Table 3). It is known that CAT activity in the liver is among the highest compared to other tissues^50^, so it is possible that it is already high enough to meet the needs of I/R-induced increased oxidative stress completely. The observed rise in hepatic GST activity in animals from I/R group (Table 3) represents a way of antioxidative protection against lipid hydroperoxides^51^, in agreement with the observed increase in the hepatic level of lipid peroxidation in the same group of animals (Table 3).

**Table 3:** The activities of liver copper-zinc superoxide dismutase (CuZnSOD, U/g tissue), manganese superoxide dismutase (MnSOD, U/g tissue), catalase (CAT, μmol H_2_O_2_/min/g tissue), glutathione peroxidase (GSH-Px, μmol NADPH/min/g tissue), glutathione reductase (GR, nmol NADPH/min/g tissue) and glutathione S-transferase (GST, nmol GSH/min/g tissue), as well as liver concentrations of glutathione (GSH, nmol/g tissue), sulfhydryl groups (SH, nmol/g tissue), and lipid peroxides (TBARS, nmol/g tissue).

	S	S + M	I/R	I/R + M
CuZnSOD	6352 ± 157	6113 ± 121	3548 ± 171^a^	5967 ± 133.164^b^
MnSOD	3358 ± 74	4599 ± 134^a^	1297 ± 48^a^	2014 ± 37^ab^
CAT	76,959 ± 2015	91,565 ± 2,706^a^	77,272 ± 2,723	147,865 ± 5,002^ab^
GSH-Px	72,172 ± 4408	74,382 ± 1,879	55,901 ± 3,113^a^	14,320 ± 4,243^ab^
GSH	4037 ± 241	6449 ± 209^a^	1513 ± 99^a^	3645 ± 95^ab^
GR	7852 ± 275	8135 ± 377	17,126 ± 397^a^	20,765 ± 654^ab^
GST	27,369 ± 6190	29,007 ± 4,983	37,262 ± 5,175^a^	33,139 ± 6,919^ab^
SH	4988 ± 192	6207 ± 260^a^	5076 ± 195	7499 ± 371^ab^
TBARS	1.242 ± 0.036	1.202 ± 0.035	1.400 ± 0.050^a^	1.180 ± 0.029^b^

Experimental group abbreviations: *S* sham-operated rat group, *S + M* sham-operated + meldonium rat group, *I/R* ischemia/reperfusion rat group, *I/R + M* ischemia/reperfusion + meldonium rat group. The number of animals per experimental group: n = 8. The data are given as mean ± standard error of the mean. Minimal significant level: p < 0.05. Significantly different: ^a^in respect to S group; ^b^in respect to I/R group.

In I/R + M group, meldonium restores hepatic antioxidative defence mostly through reverse changes relative to those induced by I/R, i.e., through the CuZnSOD and MnSOD activity increases by 50%, the GSH-Px activity and GSH content increase 2.5-fold, and the GST activity decreases by 10% (Table 3). Unexpectedly, meldonium pre-treatment also doubled the CAT activity in comparison to the I/R group, probably because of a remarkable (fivefold) increase of p-Nrf2 expression (Fig. 1C). Due to its peroxisomal location^52^, catalase is a common marker of the organelles number and localisation^53^. Peroxisomes catalyse a broad range of substrates, mainly related to lipid metabolism. As we pointed earlier, meldonium redirects long-chain FAs transport to peroxisomes^12^, thus increasing peroxisomal FAs oxidation and the peroxisomes density by FAs interaction with the peroxisome proliferator-activated receptor alpha^54^. In this way, the observed increased of CAT activity in I/R + M group may reflect the changes in peroxisomes density, as a kind of molecular evidence of meldonium physiological effects.

It should be noted that an increase in hepatic MnSOD, CAT, and GSH observed in animals from S + M group (Table 3) was not linked to the Nrf2 expression level, which remained unchanged in comparison to the sham-operated controls (Fig. 1C). The inhibitory effect of meldonium on the hepatic FAs oxidation could be a possible explanation, as presented in Tables 1 and 2. Main sites of the mitochondrial ROS production are electron transport chain Complexes I and III, whose electron-donating centres, once when they reach highly reduced state, enable an electrons “leak”, and consequent superoxide anion radical production. The redox status of those centres depends, among others, on the rate of chain electron flow, whose decrease leads to the centres higher reduced state, and thus increased oxidative stress^55^. The catabolic intermediates and the by-product of long-chain FAs oxidation are known to slow-down the chain electron flow rate^56^, and inhibit the activity of several matrix enzymes involved in maintenances of mitochondrial redox balance and mitigation of ROS formation^57^. By redirecting long-chain FAs transport to peroxisomes, meldonium prevents the mitochondrial accumulation of these FAs intermediates^1,3^ and reduces the mitochondrial ROS formation, whose ratio asymptotically increases with the FAs length^58^. In this way, meldonium decreases the risk of long-chain FAs mediated mitochondrial oxidative stress by a mechanism that is Nrf2 independent.

Another proof that meldonium prevents I/R-induced oxidative stress are the changes in the level of lipid peroxidation and the thiol groups concentration (Table 3). The concentration of the SH groups can be considered as a degree of the cell reduced state, which is why its increase indicates reduced oxidative stress^59^. The level of lipid peroxidation, on the other hand, can be considered as a general indicator of oxidative stress. Our results show that I/R, while not affecting the SH group concentration, increases the level of lipid peroxidation, which meldonium pre-treatment decreases (Table 3). In addition, meldonium also increases the SH concentration in the liver of both the I/R and sham-operated rats, confirming the antioxidative effect of meldonium.

The ratio between AA and DHA is an important marker of tissue antioxidative defence. The redox reactions of vitamin C start with the single-electron oxidation of AA into mono-dehydroascorbate radical (MDHA), which further goes through the process of dismutation to DHA plus AA^60^. Both MDHA and DHA are reduced back to ascorbate by several enzymatic systems in a process referred to as vitamin C recycling^61^. For that reason, the decrease in AA/DHA ratio indicates the attenuation of tissue antioxidant defence and increased oxidative stress. In contrast, its increase indicates improved tissue antioxidant defence and decreased oxidative stress. Thus, the fact that I/R decreases hepatic AA/DHA ratio, increased by meldonium pre-treatment in both sham-operated and I/R animals (Table 4), confirms the role of I/R in promotion of oxidative stress, and its prevention by meldonium.

**Table 4:** The ascorbic/dehydroascorbic acid ratio (AA/DHA, % of sham) in the serum, liver, and adrenal glands, and adrenal noradrenaline and adrenaline content (µg/mg tissue).

	S	S + M	I/R	I/R + M
Liver	100.00 ± 4.58	185.80 ± 10.42^a^	57.67 ± 4.42^a^	139.39 ± 7.04^ab^
Serum	100.00 ± 4.44	197.26 ± 17.02^a^	24.22 ± 2.67^a^	50.25 ± 2.17^ab^
Adrenal	100.00 ± 12.87	290.09 ± 19.38^a^	187.09 ± 13.74^a^	176.34 ± 8.46^a^
Noradrenaline	0.340 ± 0.011	2.185 ± 0.105^a^	0.098 ± 0.014^a^	0.088 ± 0.013^a^
Adrenaline	1.244 ± 0.048	1.068 ± 0.061	0.789 ± 0.076^a^	0.617 ± 0.058^a^

Experimental group abbreviations: *S* sham-operated rat group, *S + M* sham-operated + meldonium rat group, *I/R* ischemia/reperfusion rat group, *I/R + M* ischemia/reperfusion + meldonium rat group. The number of animals per experimental group: n = 8. The data are given as mean ± standard error of the mean. Minimal significant level: p < 0.05. Significantly different: ^a^in respect to S group; ^b^in respect to I/R group.

The changes in hepatic AA/DHA ratio were mapped to the serum (Table 4), as the major body depot of vitamin C, which primarily depends on ascorbate synthesis in the liver^62^. In contrast to liver and serum, in adrenal glands, the AA/DHA ratio was increased in all experimental groups (Table 4). While in S + M group it can be explained as a consequence of identical changes in liver and serum, in I/R and I/R + M groups, it is probably the result of impaired adrenal catecholamine synthesis. Ascorbic acid is an important cofactor for noradrenaline synthesis, during which it undergoes oxidation^17^. Therefore, the decrease in NA synthesis and its further conversion to AD may preserve ascorbic acid oxidation causing an AA/DHA ratio increase, in accordance with the data showing adrenal catecholamine decrease in animals from I/R and I/R + M groups (Table 4). However, it remains unclear why a sevenfold increase of NA in S + M rat group causes an even higher increase in the AA/DHA ratio in comparison to I/R and I/R groups.

**Figure 2:**
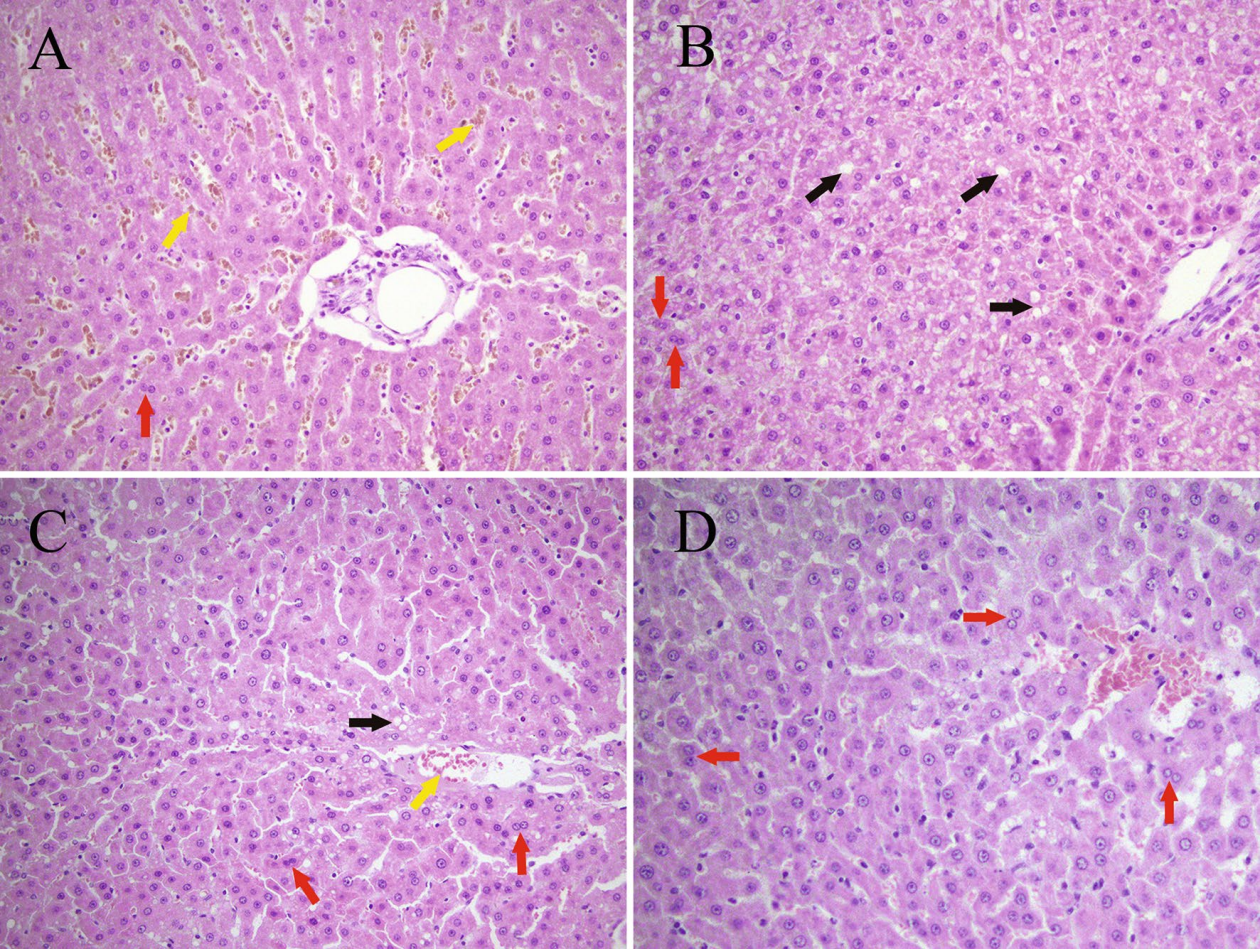
Liver histology analysis (H&E stain, 40 ×). Experimental group abbreviations: (**A**) sham-operated (S) rat group, (**B**) sham-operated + meldonium (S + M) group, (**C**) ischemia/reperfusion (I/R) rat group, (**D**) ischemia/reperfusion + meldonium (I/R + M) rat group. Yellow arrow—erythrocytic extravasation into sinusoidal spaces; black arrow—vacuolar degeneration; red arrow—binuclear hepatocytes.

As we discussed earlier, meldonium exerts its antioxidative effect through both p-Nrf2 dependent and independent pathways. However, the role of meldonium in the regulation of nitric oxide (NO) production can also be considered as the way of meldonium antioxidative action. NO is produced by three isoforms of nitric oxide synthases (NOS): endothelial (eNOS), neuronal (nNOS), and inducible (iNOS). While many of NO functions are protective or anti-inflammatory, the role NO in I/R is considered to be detrimental^63^. We did not measure iNOS expression nor NO concentration in this experiment, but it is to be expected that both will be increased in the I/R group as a result of the increased p-NF-κB p65 expression and decreased in the I/R + M group as a result of decreased p-NF-κB p65 expression (Fig. 2). Besides, it seems that meldonium can also lower NO tissue concentration by an unknown mechanism which does not depend on the NOS activity, nor the p-NF-κB p65 expression^64^.

Aspartate aminotransferase and alkaline phosphatase are enzymes present in a variety of tissues, including the liver, brain, pancreas, heart, kidneys, lungs, and skeletal muscles^65^. Consequently, if any of these tissues are damaged, AST and ALP will be released into the bloodstream. Our results showed that I/R causes 16-fold increases in ALT and AST, and a 1.6-fold increase in ALP activity (Table 5), following the literature data showing that I/R causes a much greater increase in serum ALT and AST activity than in ALP^66^. While in the sham-operated animals meldonium decreases the activity of all three enzymes, in the I/R animals, it lowers activity only of ALT. An explanation could be the fact that ALT, with the highest activity in hepatocytes and low in other tissues, represents a much better marker of hepatocellular injury than AST or ALP, which share similar activity between liver and numerous non-hepatic tissues.

**Table 5:** The serum alanine aminotransferase (ALT), aspartate aminotransferase (AST), and alkaline phosphatase (ALP) activities (IU/L).

	S	S + M	I/R	I/R + M
ALT	46.33 ± 2.49	30.25 ± 1.05^a^	775.21 ± 51.47^a^	546.75 ± 31.77^ab^
AST	76.17 ± 4.05	62.60 ± 2.59^a^	1,311.00 ± 184.36^a^	1,107.36 ± 99.67^a^
ALP	71.83 ± 3.09	49.40.25 ± 3.33^a^	116.50 ± 10.50^a^	112.00 ± 8.83^a^

Experimental group abbreviations: *S* sham-operated rat group, *S + M* sham-operated + meldonium rat group, *I/R* ischemia/reperfusion rat group, *I/R + M* ischemia/reperfusion + meldonium rat group. The number of animals per experimental group: n = 8. The data are given as mean ± standard error of the mean. Minimal significant level: p < 0.05. Significantly different: ^a^in respect to S group; ^b^in respect to I/R group.

Experimental group abbreviations: *S* sham-operated rat group, *S + M* sham-operated + meldonium rat group, *I/R* ischemia/reperfusion rat group, *I/R + M* ischemia/reperfusion + meldonium rat group.

Based on the result of liver histology analysis, it can be concluded that in sham-operated animals meldonium worsens the histological profile of the liver, the proof of which is the higher Suzuki histological score of samples from the S + M group in comparison to samples from the S group (Table 6). While samples from the S group show mild to moderate disorganisation of the hepatic cords, intensive interstitial oedema, mild erythrocytic extravasation into sinusoidal spaces (yellow arrow), intensive and diffuse parenchymal vacuolisation with the irregular nuclear contours, chromatin condensation, pyknosis, and nuclear dust in many hepatocytes, and a lack of presence of binuclear hepatocytes (red arrow) (Fig. 2A), samples from the S + M group show an intensive hepatic cords disorganisation associated with marked interstitial oedema, diffuse and pronounced cytoplasmatic vacuolisation in the form of microvascular degeneration (black arrow), regular nuclei with no evidence of chromatin condensation, pyknosis, nuclear dust, and focally presented binuclear hepatocytes (red arrow) (Fig. 2B). Although vacuolar degeneration seen in S + M group represents an unspecific marker of liver injury, a possible role of the meldonium liver biotransformation as a potential cause of it can be discussed^15^. However, it should be stressed that more prominent and intense cytoplasmic changes in the S + M group are still reversible, without nuclear damage, in contrast to histological changes in samples from S group which indicate cell damage without regeneration^67^.

**Table 6:** Liver Suzuki histological score (0–4) based on the sinusoidal congestion, vacuolization of hepatocyte cytoplasm, and parenchymal necrosis.

	Congestion	Vacuolization	Necrosis	Score
S	Moderate	Moderate	≤ 30%	3
S + M	Severe	Severe	≤ 60%	4
I/R	Mild	Mild	≤ 30%	2
I/R + M	Mild	Minimal	Single cells	1

Experimental group abbreviations: *S* sham-operated rat group, *S + M* sham-operated + meldonium rat group, *I/R* ischemia/reperfusion rat group, *I/R + M* ischemia/reperfusion + meldonium rat group.

By comparing the samples from the I/R and I/R + M groups, it can be concluded that the prevalence and intensity of liver regeneration is significantly higher in the animals pre-treated with meldonium^68^, proved with the lower Suzuki histological score in samples from the I/R + M group in comparison to samples from the I/R group (Table 6). The samples from the I/R + M group show mild interstitial oedema and moderate microhemorrhages, rare focal hepatocytes vacuolisation, microvesicular degeneration, many binuclear hepatocytes as a sign of reparation (red arrow), and very rare hepatocytes with paler nuclei and cytoplasmatic granulation (Fig. 2D), in contrast to the samples from the I/R group which show mild disorganisation of hepatic cords, followed by mild interstitial oedema, focal erythrocytic extravasation into cystic sinusoidal spaces (yellow arrow), hepatocytes enlargement with focal cytoplasmatic vacuolisation localised around micro-bleedings (black arrow), pyknotic nuclei and chromatin condensation as a sign of necrosis, and diffusely presented binuclear hepatocytes (red arrow) (Fig. 2C).

## Conclusions

**Figure 3:**
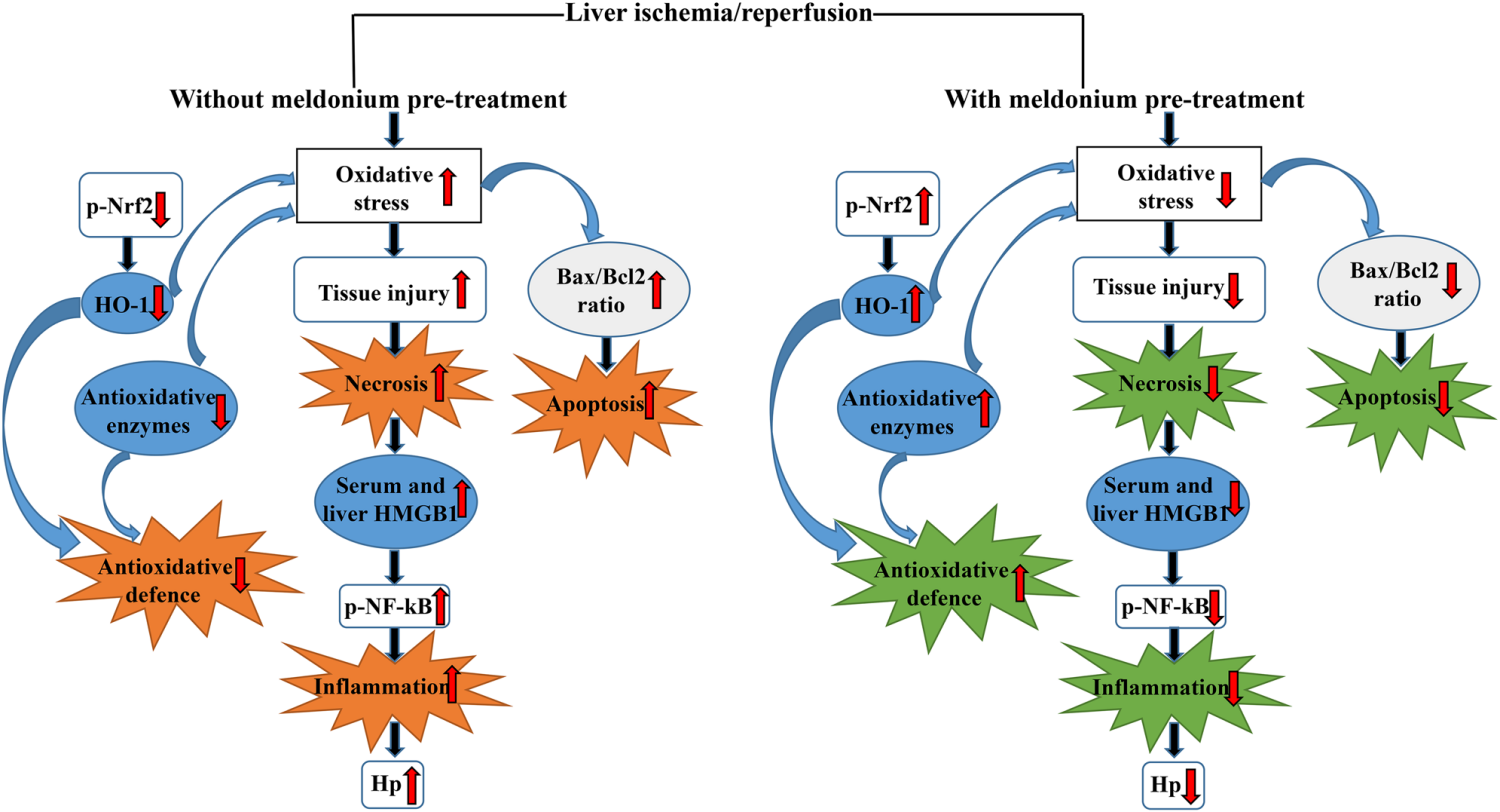
The summarised effects of meldonium pre-treatment on the liver ischemia/reperfusion. Meldonium pre-treatment improves the antioxidative defence through the increased hepatic activation of the nuclear factor erythroid 2-related factor 2 (p-Nrf2) and consequently hepatic haem oxygenase 1 (HO-1) expression, and activity of endogenous antioxidant enzymes. Thereby inhibits oxidative burst, tissue injury and cell necrosis, manifested by decreased expression of serum and liver high mobility group box 1 (HMGB1). Meldonium also reduces inflammatory cascade, as evident by decreased activation of the nuclear factor kappa-light-chain-enhancer of activated B cells (p-NF-kB p65) by phosphorylation along with decreased haptoglobin (Hp) expression. Simultaneously, meldonium attenuates apoptosis by decreasing the ratio of pro-apoptotic Bax and anti-apoptotic Bcl-2.

The main effects of meldonium and I/R in our experiment are summarised in Fig. 3. According to the presented results, it can be concluded that meldonium protects the liver against I/R-induced injury in many aspects, including anti-apoptotic, anti-necrotic, anti-inflammatory, and antioxidative. Some of these effects are the result of the observed changes in p-Nrf2 expression level, while others could be explained by meldonium-caused inhibition of long-chain fatty acids β oxidation and shift of energy production to glycolysis. Although it remains to be tested in clinical trials, the use of meldonium in preoperative management of patients subjected to surgical resection or liver transplantation represents an entirely new therapeutic approach in this field. This is especially true in relation to the efficacy of pre-treatment as the therapeutic approach since the use of drugs once when an injury occurs quickly reaches the viability threshold limit. Having all in mind, it can be concluded that meldonium might represent a protective agent against I/R-induced liver injury, with a clinical significance.

## Materials and methods

### Animals and treatments

The experiment was performed in accordance with the Directive 2010/63/EU of the European Parliament. Upon request of the National legislation, animal procedures were approved by the Veterinary Directorate of the Ministry of Agriculture, Forestry and Water Management, License number 323-07-07137/2018-05.

Thirty-two male adult Wistar rats, with the average weight of 352.73 ± 4.08 g, were randomly divided into four groups with eight animals per group (i.e. sample size per group: n = 8 animals). Animals were housed two per cage and kept on the temperature of 22 ± 1 °C under 12-h light/dark regime. The experiment lasted for four weeks, with ad libitum access to standard rat food (Veterinary Institute, Subotica, Serbia), and tap water with or without dissolved meldonium. Animals were divided into groups as follows: sham-operated animals that drunk water without meldonium for four weeks before the surgical procedure (S group); sham-operated animals that drunk water with meldonium for four weeks before to surgical procedure (S + M group); I/R-operated animals that drunk water without meldonium for four weeks before the surgical procedure (I/R group); and I/R-operated animals that drunk water with meldonium for four weeks before the surgical procedure (I/R + M group).

Meldonium (Shenzhen Calson Bio-Tech Co., Ltd., China) concentration in water was adjusted weekly to achieve daily animals’ consumption of around 300 mg/kg b.m. Based on the whole experiment data, the average meldonium consumption was 304.29 ± 3.18 mg/kg b.m./day in S + M group, and 298.36 ± 4.11 mg/kg b.m./day in I/R + M group of animals.

### Operative procedures

The model of partial liver I/R with 60 min of ischemia, followed by 4 h of reperfusion was performed using a previously described protocol^69^. Rats fasted for 16 h preceding the operation but were provided access to drinking water. The rats were anaesthetised with an intraperitoneal injection of 120 mg/kg sodium thiopental (Thiopental, Nycomed Pharma GmbH, Unterschleissheim, Germany) and placed supinely on a heating pad (37.5 ± 1 °C). The saline infusion was provided during the whole experiment (8 ml/kg/h before reperfusion, and 2 ml/kg/h during reperfusion), and anaesthesia was maintained by sodium thiopental (10 mg/kg, i.v.). After achieving surgical anaesthesia, the abdominal wall was opened, and porta hepatis and the portal triad (portal vein, hepatic artery, and biliary duct) identified. An atraumatic clamp was placed distal to the branch for the right lobe and used to prevent blood supply to the left, median, and caudate lobes of the liver. Occlusion was verified visually by the change in liver colour to a paler shade and upon reperfusion to a blush. After 60 min of ischemia, the clamp was removed, and the liver was subjected to reperfusion. At the end of the reperfusion period, anaesthetised rats were euthanised by cervical dislocation. Sham-operated rats underwent the same protocol without vascular occlusion.

### Tissue sampling

Immediately after the euthanasia, blood was collected, serum prepared^70^, and stored at − 80 °C until the analysis. The liver and adrenals were isolated and dissected out within 3 min, placed in ice-cold 155 mmol NaCl, and washed with the same solution. One part of the liver was placed in formaldehyde for further histological analysis. The tissue samples for the rest of the analysis were stored at − 80 °C until the analysis.

### Biochemical analysis

The lactic acid, carnitine, L-ascorbic acid, phosphoric acid, meta-phosphoric acid (MPA), sodium phosphate monobasic dihydrate, dithiothreitol (DTT), ethylene glycol tetra-acetic acid (EGTA), DL-Noradrenaline hydrochloride (NA), perchloric acid and magnesium chloride (MgCl_2_ × 5H_2_O) were purchased from Sigma Aldrich. The glucose was purchased from Tokyo Chemical Industry (TCI Europe, Belgium). The glycerol was included in Sugar Alcohols Kit by Supelco (Sigma Aldrich). Potassium hydroxide, sodium hydroxide, sodium acetate trihydrate, and acetonitrile were purchased from Merck (Darmstadt, Germany). Ammonium formate was supplied by Fisher Scientific, formic acid (49–51%) by Fluka Analytical, and methanol by J.T. Baker. All chemicals were of an analytical grade of purity. Ultra-pure water was obtained via a BlueClearRO600P reverse osmosis system with integrated BlueSoft07-MB mixed bed salt remover (Euro-Clear Ltd., Hungary).

Tissue samples (50 mg) were pulverised in a tissue grinder with 2 ml of ultra-pure deionised water. After the extraction at 0 °C in the ultrasound bath, the samples were centrifuged at 12,000 rpm. The supernatant was transferred in a standard flask and diluted with ultra-pure water to the 10 ml mark. The sample solutions were kept at − 80 °C until analysis. The concentration of lactic acid, glucose, carnitine, glycerol, and fatty acids was determined as described earlier^11,71^.

The quantification of lactic acid, glucose, and glycerol was done by ion chromatography, using Dionex ICS-3000 chromatographic system and IonPac AS15 Analytical, 4 × 250 mm (P/N 053940), and IonPac AG15 Guard, 4 × 50 mm (P/N 053942) separation columns in case of lactic acid, and the CarboPac PA100 pellicular anion-exchange column (4 × 250 mm) in case of glucose and glycerol (Dionex, Sunnyvale, CA, USA). The detection was performed using a gold electrode as the working, and an Ag/AgCl as the reference electrode.

For the lactic acid, a mobile phase flow rate was set at 1 ml/min, with the following gradient: 0–15 min: 10 mM KOH; 15–25 min: 10–45 mM KOH; 25–26 min: 45 mM KOH; 26–31 min: 45–10 mM KOH; and 31–36 min: 10 mM KOH.

For the glucose, a mobile phase flow rate was set at 0.7 ml/min, with the following gradient: 0–5 min: 15% A, 85% C; 5.0–5.1 min: 15% A, 2% B, 83% C; 5.1–12 min: 15% A, 2% B, 83% C; 12–12.1 min: 15% A, 4% B, 81% C; 12.1–20 min: 15% A, 4% B, 81% C; 20–20.1 min: 20% A, 20% B, 60% C; and 20.1–30 min: 20% A, 20% B 60% C (were A represents 600 mM sodium hydroxide, B 600 mM sodium acetate, and C ultrapure water).

For the glycerol, a mobile phase flow rate was set at 0.5 ml/min, with the following gradient: 0.0–10 min: 10% A; 10.0–15.0 min: 10%–50% A; 15.0–15.1 min: 50%–80% A; and 15.1–20.0 80% A (were A represents 100 mM NaOH).

The quantification of carnitine was done by thin-layer chromatography, using CAMAG chromatographic chamber and Linomat 5 plates (Camag, Switzerland), RP-18 silica (Art. 5559, Merck, Germany), and acetonitrile–water binary mixture (1:1 v/v). The plates were scanned by CAMAG TLC Scanner 3 at 260 nm.

The quantification of triglycerides was done by planar chromatography. The 2 µl of lipid extract was applied to the 20 × 10 cm HPTLC silica gel plates (Art. 105641, Merck) as 6 mm band by using Automatic TLC sampler 4 (ATS4, Camag, Switzerland). Development of the plates was performed in CAMAG twin trough chamber using 4 mobile phases:chloroform:methanol:acetic acid (90:10:1, v/v/v) up to 25 mm, *n*-hexane:diethyl-ether:acetone (60:40:5, v/v/v) up to 70 mm, *n*-hexane:diethyl-ether up to 85 mm, and 100% n-hexane up to 90 mm. For derivatisation, the mixture of concentrate sulphuric acid and methanol (1:9, v/v) was used. Derivatised plates were heated at 110 °C on TLC Plate Heater III (Camag, Switzerland) until chromatographic zones become visible, followed by scanning in CAMAG TLC scanner 3 equipped with Linomat 4.

The FAs were extracted from the tissue by Folich modified method^72^. The analysis was performed on Focus GC coupled with the PolarisQ mass spectrometer (Thermo Fisher, USA), using helium (1 ml/min) as the carrier gas. The oven temperature program was as follows: the initial temperature 50 °C (1 min), then 25 °C/min to 200 °C, and immediately 3 °C/min to 230 °C (held for 18 min). The injector was in split mode (50:1), while the injector, transfer line, and ion source temperatures were 250 °C, 260 °C and 260 °C, respectively. It was investigated the concentration of 18 fatty acids: myristic (tetradecanoic, C14:0), pentadecylic (pentadecanoic, C15:0), palmitic (hexadecanoic, C16:0), palmitoleic (*cis*-Δ9 hexadecenoic, C16:1), margaric (heptadecanoic, C17:0), heptadecenoic acid (*cis*-Δ10 heptadecenoic acid, C17:1), stearic (octadiecanoic, C18:0), oleic/elaidic (*cis*- and *trans*-Δ9 octadecenoic, C18:1), linoleic/linolelaidic (*cis*- and *trans*-Δ9,12 octadecenoic, C18:2 c + t), linolenic (*cis*- and *trans*-Δ9,12,15 octadecadienoic, C18:3) gondoic acid (*cis*-11 eicosenoic acid C20:1), eicosadienoic (*cis*-11,14 eicosadienoic acid, C20:2), dihomo-γ-linolenic (*cis*-Δ8,11,14 eicosatrienoic acid, C20:3) dihomo-α-linolenic (*cis*,*cis*,*cis*-Δ11,14,17 eicosetrienoic, c20:3), behenic (docosanoic, C22:0), cervonic (*cis*,*cis*-Δ13,16 docosadienoic, C22:1), and euric (*cis*,*cis*,*cis*,*cis*,*cis*,*cis*-Δ4,7,10,13,16,19 docosahexaenoic, C22:6). Under applied chromatographic conditions, cervonic and euric acids coelute at the same retention time, so their concentrations were calculated as the sum. The same was the case for *cis*- and *trans*- forms of C18:2, so that the results were actually presented as the 16 fatty acids analysis.

The serum, liver, and adrenal concentration of AA and DHA was measured according to the method of Nováková et al.^73^. The tissue samples were homogenised by Ultra-Turrax homogeniser (IKA, China) in 10% MPA (1 mg:10 µl), and sonicated 3 times for 10 s. Serum samples were mixed with 10% MPA. Samples were centrifuged for 30 min at 18,000 rpm (4 °C), and the obtained supernatants were used for the analysis of native AA concentration. Total vitamin C concentration was analysed by converting sample DHA to AA with the 300 µL DTT solution in phosphate buffer (30 min. at 4 °C) and using 10% MPA for the re-acidification of samples. The data were obtained by UltiMate 3000 HPLC system, using Acclaim Polar Advantage II, C18 (3 µm) HPLC column, and the RS electrochemical detector with the glassy carbon working electrode (Thermo Fisher, USA). The mobile phase (160 mM phosphate buffer, pH 3.0) was pumped for 10 min in an isocratic flow of 800 µl/min. The applied potential was 600 mV, and the separation temperature was 25 °C.

The concentration of adrenaline (AD) and noradrenaline (NA) was performed according to the method of Stefanovic et al.^74^ The catecholamines stock standard (1 mg/ml of methanol) was diluted by DEPROT solution (2% EGTA; 0.1 N HClO_4_; 0.2% MgCl_2_) in order to get the working standard. Adrenal glands were homogenised by Ultra-Turrax homogeniser (IKA, China) in DEPROT (1 mg:10 µl), and sonicated 3 times for 10 s. The data were obtained using the same HPLC system like the one described in the case of vitamin C determination. The mobile phase (100 mM ammonium formate as an A solution, and the methanol as a B solution) was pumped at a flow rate of 500 µl/min with the following gradient: 98% A and 2% B solution mix was used for the run, then B solution rose to reach 80% in the 13^th^ minute starting from the minute 9, and the column was re-equilibrated with the initial mixture of mobile phase solutions (2% of A and 98% of B solution) starting from the 18th minute until the end of the run (25th min.). The applied potential was 850 mV, and the separation temperature was 25 °C.

Activities of ALT, AST and AP in serum were measured by Roche Cobas C501 Chemistry analyser, using ALTL, ASTL, and ALP2L reagent cassette (Roche Diagnostics, F. Hoffmann-La Roche Ltd, Switzerland).

### The determination of liver oxidative stress biomarkers

All chemicals were purchased from Sigma Aldrich. The liver samples were minced and homogenised in 10 volumes of 25 mmol/l sucrose containing 10 mmol/l Tris–HCl, pH 7.5 at 1,500 rpm using Ultra-Turrax homogeniser (IKA-Werke, Staufen, Germany) at 4 °C. Homogenates were centrifuged at 4 °C at 100,000×*g* for 90 min, sonicated for 30 s at 10 kHz on ice (Bandeline Sonopuls HD 2070), and centrifugated in a Beckman ultracentrifuge at 100,000×*g* for 90 min at 4 °C.

Enzyme activity in the supernatant samples was measured in triplicates using Shimadzu UV-1800 spectrophotometer and a temperature-controlled cuvette holder. Total SOD activity was measured by the method based on the capacity of SOD to inhibit the autoxidation of adrenaline to adrenochrome^75^, and the same method was applied for the MnSOD measurement after pre-incubation with 8 mmol/l KCN^76^. CAT activity was measured as the rate of the H_2_O_2_ decomposition at 240 nm^77^. GSH-Px activity was determined by measuring the oxidation of nicotinamide adenine dinucleotide phosphate (NADPH) with t-butyl hydroperoxide at 340 nm^78^. GR activity was determined by measuring NADPH oxidation at 340 nm in the presence of GSSG^79^.

GST activity was measured at 340 nm in a 100 mM tris buffer (pH 7.4), 1 mM GSH and tissue homogenate reaction mixture, using 1-chloro-2,4-dinitrobenzene as a substrate^80^. GSH content was measured by the method based on the enzymatic cycling of the GSSG to GSH by GR and NADPH, using 2-vinylpyridine as the GSH masking agent^81^. SH groups concentration was measured as the 5-thio-2-nitrobenzoic acid absorption at 412 nm, resulting from the 5,5′-dithiobis-(2-nitrobenzoic) acid reduction by free thiol groups^82^. The lipid peroxides concentration was measured as the absorbance change at 532 nm in the reaction of sample malondialdehyde with the thiobarbituric acid as the reagent^83^.

### Western immunoblot analysis

The serum samples and liver homogenates (previously prepared for determination of oxidative stress biomarkers) were separated by 12% SDS-PAGE and transferred onto polyvinylidene difluoride membranes (Hybond-P, Amersham Pharmacia Biotech), blocked in a 0.2% Tween 20, 50 mM Tris HCl pH 7.6, and 150 mM NaCl solution containing 5% non-fat condensed milk. After the protein transfer, membranes were incubated for an hour and a half at room temperature with the following primary antibodies: Cell Signalling Technology rabbit polyclonal Bax (2772) and Bcl-2 (2876); Abcam rabbit polyclonal HMGB1 (ab 18256), Hp (ab 131236), HO-1 (ab 13243), phospho-NF-κB p65 (Ser 311, ab 194926) and β-actin (ab 8227) antibodies; and the Thermo Fisher Scientific: rabbit polyclonal phospho-Nrf2 (Ser 40, PA5-67520) antibody. The blots were probed/re-probed with Abcam horseradish peroxidase-conjugated secondary (goat anti-rabbit IgG) antibodies (ab 205718, ab 97051). Immunoreactive bands were identified by an enhanced chemiluminescence detection system (Santa Cruz Biotechnology, Santa Cruz, CA, USA), and the signals intensities were quantified using TotalLab (Phoretix, Newcastle Upon Tyne, England) electrophoresis software (ver.1.10). Actin was used as a loading control for the quantification.

### Histology analysis

Liver samples were fixated in a 4% formaldehyde, dehydrated in an increasing series of ethanol solutions (70%, 96%, and 100%), and immersed in xylene. After being embedded into paraffin wax, samples were cut into 5 µm thick sections and stained with haematoxylin and eosin (H&E stain). Using the Leica DM LS 2 type 11020518016 microscope (Leica, Wentzler, Germany), a minimum of 10 fields of each liver section were analysed. The analysis evaluated tissue organisation, trabecular arrangement, sinusoidal changes, cytoplasmatic vacuolisation, nuclear characteristics, and presence of binuclear cells. A Suzuki histological score was used for blind scoring by pathologist. According to sinusoidal congestion, vacuolization of hepatocyte cytoplasm, and parenchymal necrosis, sections were scored from 0 to 4^84^.

### Statistical analysis

The normality of data was checked with the Kolmogorov*–*Smirnov test*.* The differences between the groups were analysed through one-way ANOVA. When significant differences (p < 0.05) were found, Holm–Sidak post hoc test was performed for pairwise comparisons. All analyses and graphs were carried out in statistical package SIGMAPLOT, with the data presented as the mean ± standard error of the mean.

### Ethical statement

The authors are accountable for all aspects of the work ensuring that questions related to the accuracy or integrity of any part of the work are appropriately investigated and resolved.

## Supplementary Information

Supplementary Information.
