# Supplementary material for: The effects of meldonium on the acute ischemia/reperfusion liver injury in rats
Source: Sci Rep. 2021 Jan 14;11:1305. doi: 10.1038/s41598-020-80011-y (PMC7809046; doi:10.1038/s41598-020-80011-y)

**Supplementary Information to Figure 1**

The effects of meldonium on the acute ischemia/reperfusion liver injury in rats

Siniša Đurašević^1*^, Maja Stojković^2^, Jelena Sopta^2^, Slađan Pavlović^3^, Slavica Borković-Mitić^3^, Anđelija Ivanović^3^, Nebojša Jasnić^1^, Tomislav Tosti^4^, Saša Đurović^5^, Jelena Đorđević^1^ and Zoran Todorović^2,6^

^1^ University of Belgrade, Faculty of Biology, Belgrade, Republic of Serbia

^2^ University of Belgrade, Faculty of Medicine, Belgrade, Republic of Serbia

^3^ University of Belgrade, Institute for Biological Research “Siniša Stanković” – National Institute of Republic of Serbia, Belgrade, Republic of Serbia

^4^ University of Belgrade, Faculty of Chemistry, Belgrade, Republic of Serbia

^5^ University of Belgrade, Institute of General and Physical Chemistry, Belgrade, Republic of Serbia

^6^ University of Belgrade, University Medical Centre “Bežanijska Kosa,” Belgrade, Republic of Serbia

*** Corresponding author:**

**Siniša Đurašević, Ph.D., Associate Professor,** University of Belgrade, Faculty of Biology, **16** Studentski Trg, 11000 Belgrade, Serbia, phone/fax: (+381 11) 2639-882, e-mail: [sine@bio.bg.ac.rs](mailto:sine@bio.bg.ac.rs)

**Supplemental Information to Figure 1: Original Western blots of whole liver homogenates and serum. Boxed area indicates the cropped area used in Figure 1A, 1B and 1C.**

Figure 1. Representative immunoblots of protein expression levels: A) liver Bax/Bcl-2 ratio, and liver and serum HMGB1 expression; B) liver Hp and HO-1 expression; and C) liver p-Nrf2 and p-NF-κB p65 expression, and p-NF-κB p65/p-Nrf2 ratio. β actin was used as a loading control. Experimental group abbreviations: S – sham-operated rat group; S+M – sham-operated + meldonium rat group; I/R – ischemia/reperfusion rat group; I/R+M – ischemia/reperfusion + meldonium rat group.

**Figure 1A. Bax**


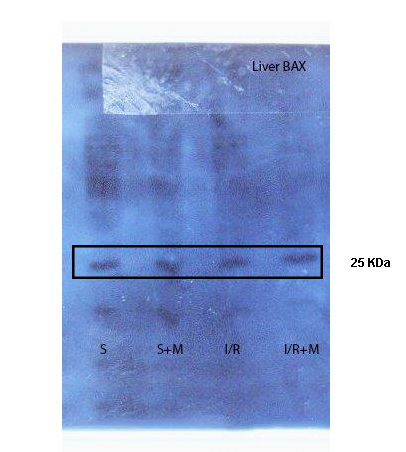


**Figure 1A. Bcl2**


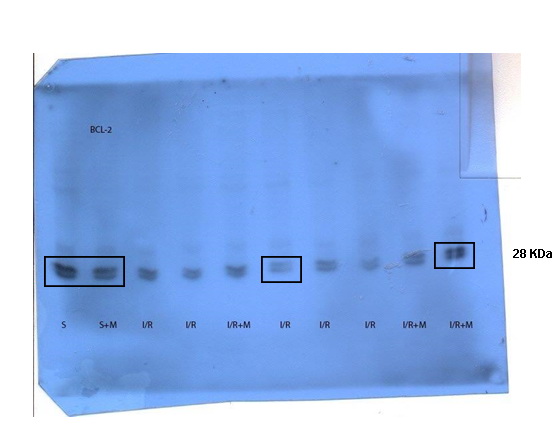


**Figure 1A. HMGB1 serum**


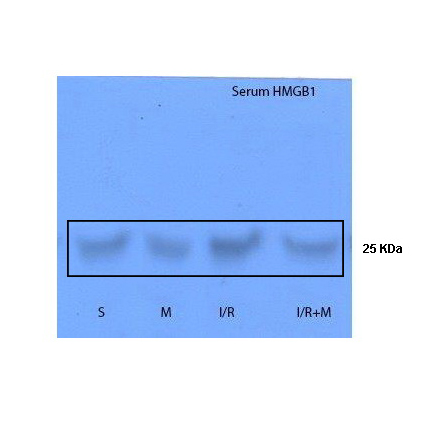


**Figure 1A. HMGB1 liver**


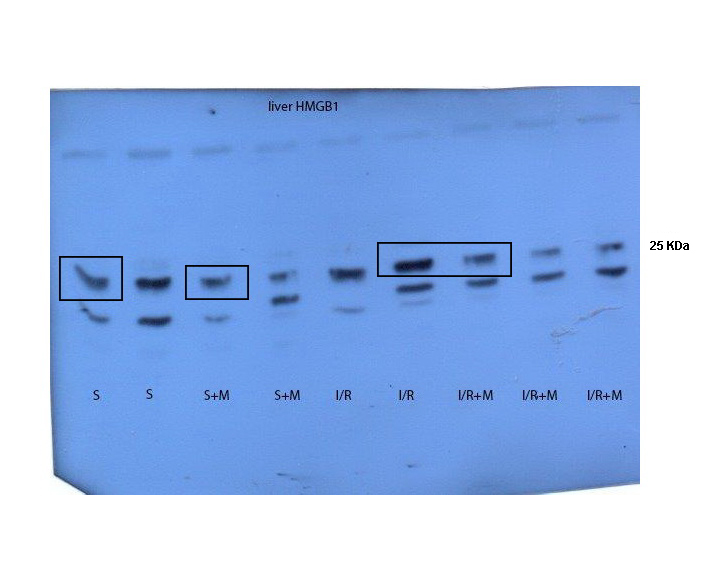


**Figure 1B. Hp**


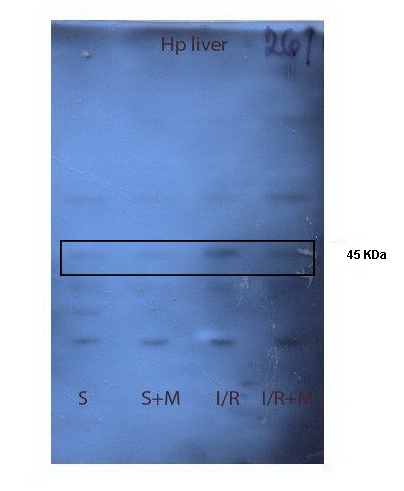


**Figure 1B. HO1**


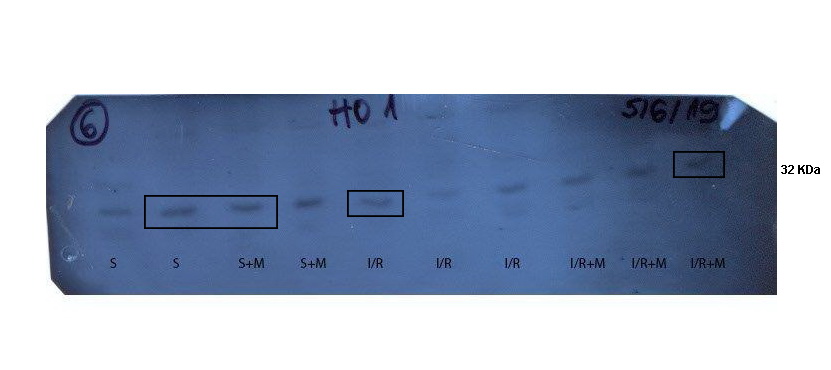


**Figure 1C. p-Nrf2**


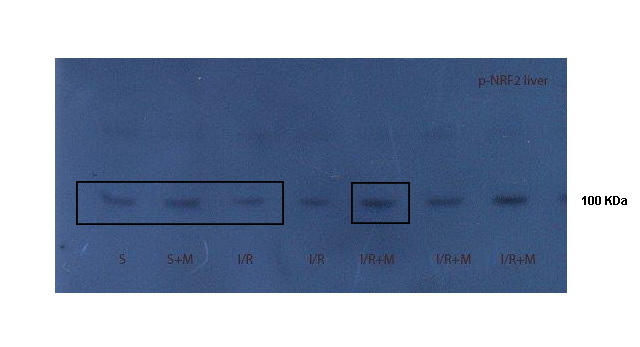


**Figure 1C. p-NFkB p65**


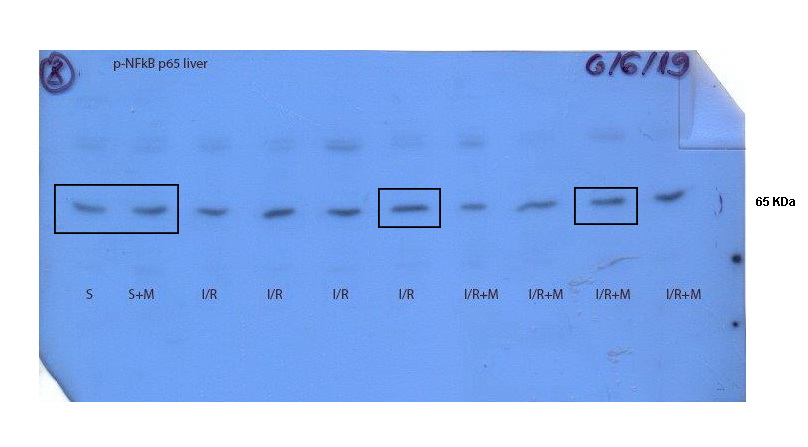


**Original Western blots of whole liver homogenates with β ACTIN antibody**

**β ACTIN 1. Original Western blot of β Actin from main Figure 1A, Bax and Bcl2; Fig. 1B, Hp and HO-1**

The protein samples indicated in boxed area (Supplement Fig. 1A, Bax and Bcl2; Fig. 1B, Hp and HO-1) were used for extra β actin Western blot presented in this manuscript**.**


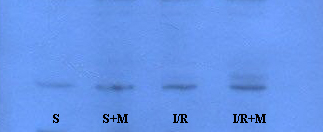


**β ACTIN 2. Original unprocessed Western blot of β Actin from main Figure 1A. Bcl2**


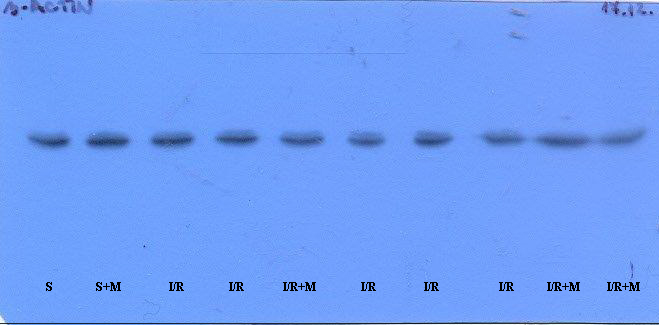


**β ACTIN 3. Original unprocessed Western blot of β Actin from main Figure 1B. HO-1**


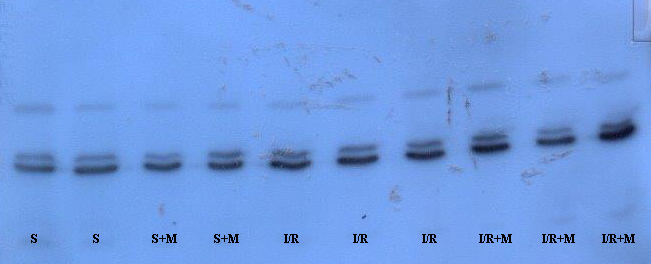


**β ACTIN 4. Original Western blot of β Actin from main Figure 1A. HMGB1 liver**

The protein samples indicated in boxed area (Supplement Fig. 1A, HMGB1) were used for extra β actin Western blot presented in this manuscript.


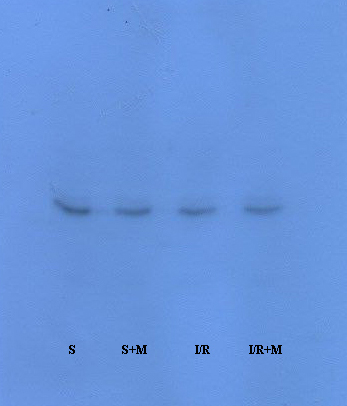


**β ACTIN 5. Original unprocessed Western blot of β Actin from main Figure 1A. HMGB1 liver**


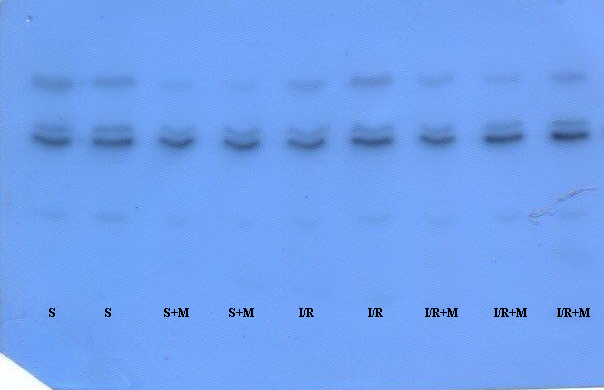


**β ACTIN 6. Original unprocessed Western blots of β Actin from main Figure 1C. p-Nrf2**


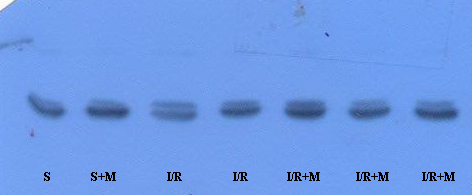


**β ACTIN 7. Original unprocessed Western blot of β Actin from main Figure 1C. p-NFkB p65**


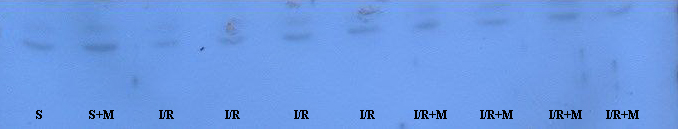

Supplement: Supplementary file 1 — Supplementary Information. [file 41598_2020_80011_MOESM1_ESM.docx]
